# A multimodal interpretable deep learning-radiomics framework for predicting lymph node metastasis following neoadjuvant chemoradiotherapy in locally advanced rectal cancer: a multicenter validation study

**DOI:** 10.1038/s41698-026-01510-1

**Published:** 2026-05-29

**Authors:** Qiurong Wei, Chenyu Zhao, Zeli Chen, Yehuan Tang, Weicui Chen, Liming Zhong, Shaowei Hu, Yuankui Wu, Wei Yang, Xian Liu

**Affiliations:** 1 Department of Radiology, The Second Affiliated Hospital of Guangzhou University of Chinese Medicine, Guangzhou, China; 2 Guangdong Provincial Key Laboratory of Medical Image Processing, School of Biomedical Engineering, Southern Medical University, Guangzhou, China; 3 Department of Radiology, First Affiliated Hospital of Guangxi Medical University, Nanning, China; 4 Department of Pathology, The Second Affiliated Hospital of Guangzhou University of Chinese Medicine, Guangzhou, China; 5 Department of Medical Imaging, Nanfang Hospital, Southern Medical University, Guangzhou, China

**Keywords:** Cancer, Computational biology and bioinformatics, Oncology

## Abstract

Accurate assessment of lymph node metastasis (LNM) following neoadjuvant chemoradiotherapy (nCRT) presents a significant clinical challenge and is essential for the management of locally advanced rectal cancer (LARC). In this multicenter study, we developed and externally tested a multimodal MRI-based framework that integrates clinical demographics, handcrafted radiomic signatures, and deep learning (DL)-derived features to predict post-nCRT lymph nodal status. This study enrolled 382 LARC patients who underwent surgery after nCRT at four centers. Post-nCRT T2-weighted (T2WI) and diffusion-weighted (DWI) MRI images were used to extract radiomic and DL-derived features of tumors. After feature harmonization and selection, a predictive model was constructed using a DL fusion network and a random forest algorithm. The model performance was evaluated across the training, validation, internal test, and external test cohorts using the area under the receiver operating characteristic curve (AUC) and decision curve analysis (DCA). Shapley Additive Explanation (SHAP) and gradient-weighted Class Activation Mapping (Grad-CAM) were used to enhance the model’s interpretability. The combined model, which included clinical, radiomics and DL-derived features, demonstrated the optimal predictive capacity, with an AUC of 0.771 in the external test dataset. This approach shows promise for noninvasively determining treatment response, prognosis, and surgical management.

## Introduction

LARC management is increasingly shifting toward individualized precision-guided strategies^1–3^. Accurate assessment of lymph node metastasis (LNM) after nCRT is crucial for surgical planning, organ preservation decisions, and long-term outcome prediction^4–7^. However, reliable nodal restaging after treatment remains challenging due to treatment-induced fibrosis, necrosis, and inflammatory alterations, which obscure typical morphological features. Additionally, current imaging criteria, such as short-axis diameter threshold and signal heterogeneity, exhibit inconsistent results across studies^8^. Functional MR sequences, such as diffusion-weighted imaging (DWI), combined with T2-weighted imaging (T2WI), provide comprehensive functional and structural information about tumors and are currently incorporated into clinical guidelines^9,10^. Nevertheless, DWI exhibits limitations in assessing post-nCRT lymph node status, including low sensitivity for small LNM and the potential for misinterpretation due to treatment-induced fibrosis. This discrepancy results in moderate diagnostic accuracy and significant interobserver variability in conventional MRI diagnosis^11,12^, highlighting the need for more objective and reliable tools for post-treatment nodal assessment.

Radiomics and deep learning (DL) have recently been rapidly developed and promoted as quantitative imaging approaches in medical imaging. Radiomics facilitates the high-throughput extraction of quantitative descriptors to characterize tumor phenotypes, with numerous studies demonstrating correlations with lymph node status and treatment response^13–16^. However, the stability and reproducibility of radiomics features are influenced by variations in imaging protocols and reconstruction parameters, which restrict their generalizability^17–19^. Although DL models have demonstrated superior predictive performance through automated feature engineering, they remain constrained by their “black-box” nature and heavy reliance on large-scale annotated training datasets^20,21^. Integrating radiomics with DL enables more comprehensive and robust feature extraction than either method alone. This synergy leverages biologically interpretable radiomic features and DL-derived features, yielding superior predictive performance^22–24^. The utilization of DL-radiomics combination has yielded promising results in the diagnosis and treatment of colorectal cancer, including preoperative prediction of KRAS mutations, differentiation from stage T2 to T3 tumors, and prediction of distant metastasis or early recurrence in LARC patients receiving nCRT^16,25–27^.

In recent years, explainable artificial intelligence (XAI) has been increasingly recognized as essential for providing transparency and interpretability to the decision-making of DL and radiomics models. Recent studies emphasize the value of interpretable AI frameworks for improving early detection, increasing diagnostic confidence, and supporting individualized clinical decision-making in fields such as imaging, pathology, surgery, and drug discovery. Therefore, integrating interpretability into multimodal predictive models is essential for building clinical trust and translational applicability^28^. To date, no studies have reported the use of an MRI-based interpretable DLR model, combined with multicenter study data to predict LNM in patients with LARC after nCRT. This study aimed to develop and validate a multimodal fusion model that integrates clinical variables, MR-based radiomics features, and DL-derived features to estimate post-nCRT LNM in patients with LARC. We emphasized model interpretability, calibration, and clinical utility to facilitate potential integration into surgical decision-support workflows. This study was conducted and reported in accordance with the TRIPOD-AI statement^29^ and the TITAN guideline^30^.

## Results

### Patient characteristics

**Fig. 1:**
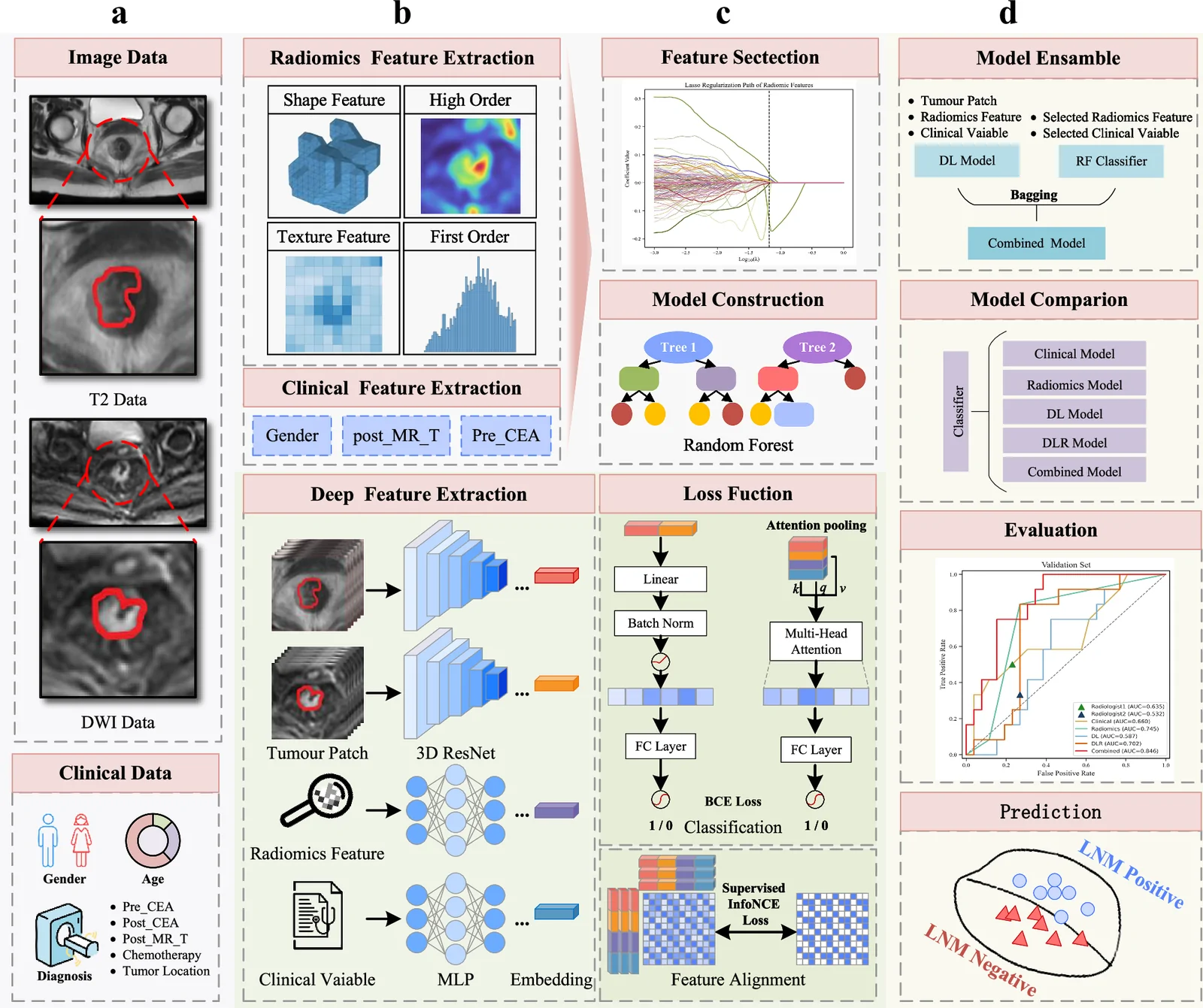
Schematic workflow of the integrated DL and radiomics framework for LNM prediction. **a** Multimodal data acquisition collection of MRI scans (post-nCRT T2 and DWI) and clinical variables (demographics and lab results). **b** Multi-stream feature extraction: extraction of radiomics (shape/texture), clinical, and DL-derived features (3D ResNet/MLP). **c** Feature selection and model training: dimensionality reduction via LASSO, Random Forest construction, and loss-based feature alignment. **d** Model ensemble and evaluation: ensemble of DL and RF models, followed by ROC performance comparison and LNM prediction. The outputs were fused via an attention-based pooling module for classification. Gradient-weighted Class activation mapping (Grad-CAM) highlights regions contributing to LNM prediction.

Ultimately, a total of 382 patients were enrolled in this study: 282 from Centers 1 and 2, 82 from Center 3, and 18 from Center 4. Figure 1 illustrates the schematic workflow of the integrated DL and radiomics framework for LNM prediction. Table 1 summarized the clinical and pathological characteristics of all 382 patients in the training/internal validation, internal test, and external test cohorts. Clinical features were generally balanced across the training/internal validation, internal test, and external test cohorts, except for chemotherapy regimen (*p* = 0.006), tumor regression grade (*p* = 0.01), and post-nCRT CEA levels (*p* = 0.005). Overall, 117 patients (30.6%) were lymph node–positive (ypN+) and 265 (69.4%) were lymph node–negative (ypN0). The cohort-specific distributions were as follows: 59/197 (30.0%) in the training cohort, 26/85 (30.6%) in the internal test cohort, and 32/100 (32.0%) in the external test cohort. Supplementary Table 1 provides detailed characteristics of Centers 3 and 4.

**Table 1:** Baseline characteristics of patients across cohorts

Characteristics	Training/internal validation cohort^a^ (*n* = 197)	Internal test cohort (*n* = 85)	External test cohort (*n* = 100)	*P* value*
Age (y, mean ± SD)	57.04 ± 10.95	56.28 ± 10.87	55.56 ± 10.82	0.535
Gender (%)				
Male	150	64	71	0.621
Female	47	21	29	
Chemotherapy regimen (%)				
Capeox	141	71	77	0.006*
Capecitabine	39	5	7	
mFOLFOX6	11	6	12	
5-fluorouracil & leucovorin	6	3	4	
Post MR_T stage (%)				
T1	0	1	0	0.749
T2	15	6	8	
T3	141	61	67	
T4	41	17	25	
Tumor location (%)				0.681
Low	70	29	30	
Middle	103	49	56	
Upper	24	7	14	
ypT stage (%)				0.207
T1	0	0	1	
T2	36	16	10	
T3	130	56	77	
T4	31	13	12	
yTRG(%)				0.01*
TRG 0	40	15	22	
TRG 1	60	33	25	
TRG 2	71	29	51	
TRG 3	26	8	2	
Histological grade (%)				
Well differentiation	18	8	11	0.620
Moderate differentiation	167	70	78	
Poor differentiation	12	7	11	
Pre-nCRT CEA level (%)				
≤5(Normal)	93	43	39	0.249
>5(Abnormal)	104	42	61	
Post-nCRT CEA level (%)				
≤5(Normal)	172	70	72	0.005*
>5(Abnormal)	25	15	28	

Chi-squared or Fisher’s exact tests were used to compare the differences in categorical variables, whereas Student’s *t* test or Mann–Whitney U test was used to compare the differences in continuous variables, as appropriate.

*LN* lymph node, *nCRT* neoadjuvant chemoradiotherapy, *CEA* carcinoembryonic antigen.

*P < 0.05.

^a^The training cohort (*n* = 197) encompasses the data used for model training and internal validation (hyperparameter optimization). The internal test cohort (*n* = 85) was a held-out set used solely for unbiased performance evaluation.

### Clinical and radiomics features selection

**Fig. 2:**
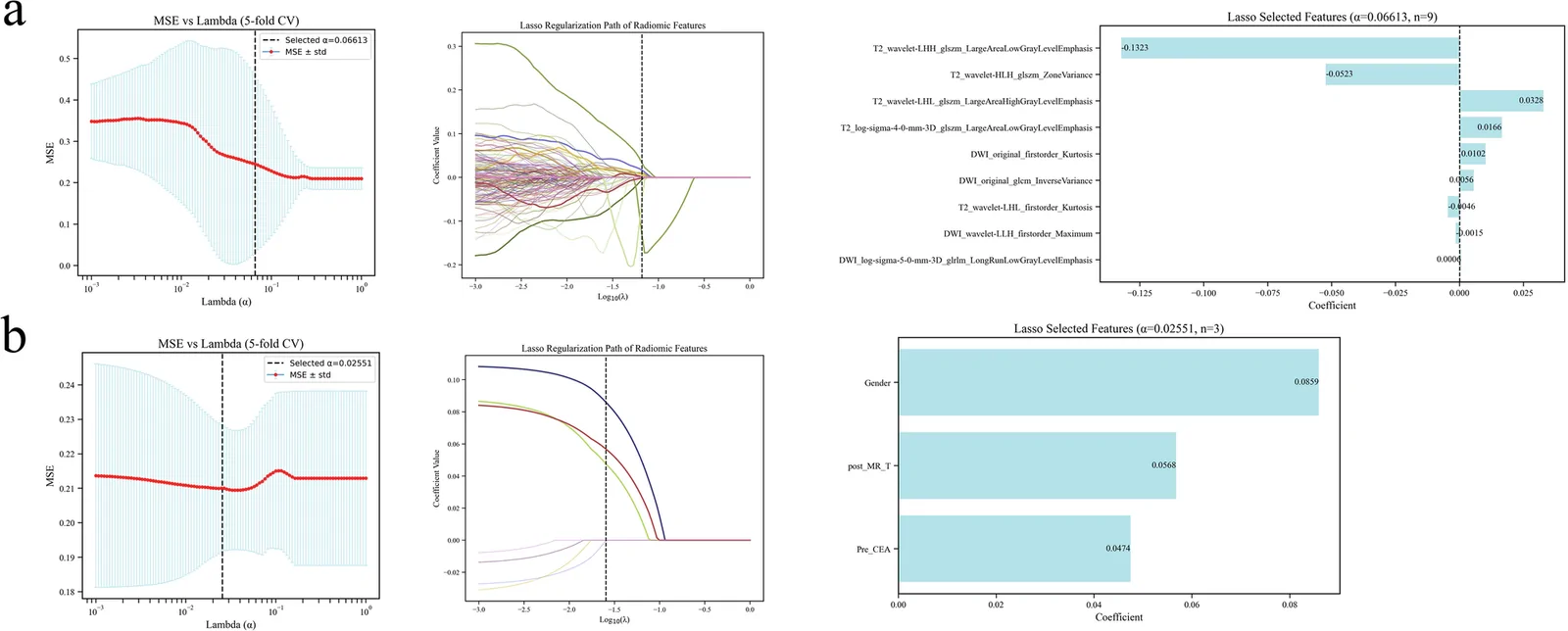
Selected clinical and radiomic predictors for LNM. The model identified significant predictors from clinical and radiomic data via LASSO with five-fold cross-validation. This process selected nine radiomic features (**a**) and three clinical features (**b**), with their corresponding coefficients.

A total of 2216 radiomic features with ICC > 0.8 were retained. Eventually, nine radiomics features were selected for model construction: four wavelet and one LoG feature from T2WI, and one wavelet, one GLCM, one first-order, and one LoG feature from DWI. Using LASSO regression, three independent clinical predictors were identified: gender, pre-nCRT CEA level, and post-nCRT MRI T stage. Figure 2 displays the selected clinical and radiomics features ranked by their coefficients. Based on these clinical variables, an RF classifier was developed to construct the clinical prediction model.

### Clinical model and radiologist performance

As shown in Table 2, the clinical model achieved AUCs of 0.752 (95% CI, 0.669–0.824) in the training cohort, 0.658 (95% CI, 0.452–0.850) in the internal validation cohort, 0.625 (95% CI, 0.488–0.748) in the internal test cohort, and 0.635 (95% CI, 0.529–0.752) in the external test cohort. For comparative analysis, the diagnostic performances of two experienced radiologists were also evaluated. Radiologist 1 achieved AUCs of 0.642, 0.637, 0.698, and 0.678 in the training, internal validation, internal test, and external test cohorts, respectively. Radiologist 2 yielded AUCs of 0.657, 0.533, 0.718, and 0.649, respectively.

**Table 2:** Predictive performance of different models across the cohorts

Models	Cohorts	AUC (95% CI)	ACC (95% CI)	SEN (95% CI)	SPE (95% CI)	PPV (95% CI)	NPV (95% CI)
Clinical model	Train	0.752(0.669–0.824)	0.612(0.535–0.679)	0.832(0.714–0.930)	0.520(0.426–0.610)	0.421(0.326–0.518)	0.881(0.797–0.951)
	Validation	0.658(0.452–0.850)	0.448(0.289–0.605)	1.000(1.000–1.000)	0.195(0.040–0.348)	0.363(0.194–0.545)	0.997(1.000–1.000)
	Internal test	0.625(0.488–0.748)	0.578(0.471–0.682)	0.733(0.562–0.895)	0.509(0.385–0.644)	0.399(0.255–0.535)	0.811(0.684–0.931)
	External test	0.635(0.529–0.752)	0.547(0.460–0.650)	0.776(0.632–0.909)	0.439(0.324–0.556)	0.393(0.273–0.517)	0.807(0.679–0.929)
Radiomics model	Train	0.999(0.996–1.000)	0.988(0.969–1.000)	0.958(0.891–1.000)	1.000(1.000–1.000)	1.000(1.000–1.000)	0.983(0.955–1.000)
	Validation	0.745(0.577–0.894)	0.762(0.605–0.895)	0.831(0.571–1.000)	0.730(0.550–0.889)	0.585(0.333–0.826)	0.905(0.750–1.000)
	Internal test	0.731(0.648–0.811)	0.660(0.553–0.765)	1.000(1.000–1.000)	0.509(0.371–0.638)	0.475(0.340–0.611)	1.000(1.000–1.000)
	External test	0.641(0.529–0.743)	0.680(0.590–0.760)	0.593(0.419–0.757)	0.721(0.606–0.816)	0.499(0.333–0.657)	0.791(0.683–0.885)
DL model	Train	0.769(0.684–0.844)	0.723(0.654–0.786)	0.678(0.538–0.808)	0.742(0.664–0.818)	0.524(0.397–0.647)	0.846(0.775–0.910)
	Validation	0.585(0.407–0.760)	0.469(0.316–0.632)	1.000(1.000–1.000)	0.225(0.074–0.400)	0.372(0.207–0.552)	0.996(1.000–1.000)
	Internal test	0.561(0.429–0.702)	0.411(0.318–0.518)	0.962(0.880–1.000)	0.166(0.079–0.270)	0.339(0.239–0.447)	0.907(0.700–1.000)
	External test	0.636(0.522–0.745)	0.471(0.380–0.570)	0.939(0.844–1.000)	0.251(0.149–0.355)	0.370(0.270–0.470)	0.897(0.739–1.000)
DLR model	Train	0.989(0.972–1.000)	0.968(0.937–0.994)	1.000(1.000–1.000)	0.955(0.914–0.991)	0.903(0.814–0.978)	1.000(1.000–1.000)
	Validation	0.701(0.521–0.862)	0.708(0.553–0.842)	0.831(0.571–1.000)	0.652(0.462–0.840)	0.522(0.286–0.765)	0.895(0.733–1.000)
	Internal test	0.709(0.595–0.813)	0.635(0.518–0.729)	0.923(0.810–1.000)	0.507(0.365–0.642)	0.454(0.320–0.583)	0.936(0.839–1.000)
	External test	0.707(0.604–0.799)	0.631(0.540–0.730)	0.844(0.708–0.967)	0.531(0.409–0.651)	0.458(0.333–0.596)	0.879(0.767–0.976)
Combined model	Train	0.999(0.998–1.000)	0.987(0.968–1.000)	1.000(1.000–1.000)	0.982(0.953–1.000)	0.959(0.896–1.000)	1.000(1.000–1.000)
	Validation	0.842(0.699–0.950)	0.813(0.684–0.921)	0.744(0.467–1.000)	0.845(0.704–0.964)	0.687(0.417–0.929)	0.877(0.731–1.000)
	Internal test	0.769(0.662–0.864)	0.728(0.624–0.812)	0.885(0.759–1.000)	0.658(0.529–0.768)	0.535(0.390–0.683)	0.928(0.844–1.000)
	External test	0.771(0.668–0.857)	0.722(0.630–0.810)	0.752(0.600–0.900)	0.708(0.597–0.813)	0.547(0.400–0.691)	0.859(0.759–0.942)
Radiologist 1	Train	0.642(0.553–0.723)	0.719(0.648–0.786)	0.452(0.304–0.595)	0.831(0.760–0.900)	0.529(0.359–0.691)	0.784(0.710–0.850)
	Validation	0.637(0.473–0.801)	0.685(0.526–0.842)	0.508(0.222–0.800)	0.767(0.591–0.920)	0.500(0.222–0.818)	0.771(0.600–0.920)
	Internal test	0.698(0.598–0.805)	0.730(0.647–0.824)	0.616(0.435–0.806)	0.781(0.679–0.883)	0.555(0.375–0.727)	0.821(0.722–0.914)
	External test	0.678(0.575–0.774)	0.661(0.560–0.750)	0.726(0.567–0.882)	0.631(0.500–0.746)	0.480(0.340–0.622)	0.831(0.717–0.931)
Radiologist 2	Train	0.657(0.573–0.740)	0.732(0.660–0.799)	0.473(0.333–0.614)	0.841(0.771–0.905)	0.555(0.395–0.718)	0.792(0.718–0.858)
	Validation	0.533(0.371–0.696)	0.606(0.447–0.763)	0.337(0.083–0.625)	0.729(0.552–0.906)	0.365(0.091–0.714)	0.705(0.522–0.871)
	Internal test	0.718(0.616–0.826)	0.742(0.659–0.835)	0.655(0.476–0.833)	0.781(0.681–0.881)	0.571(0.394–0.733)	0.836(0.740–0.929)
	External test	0.649(0.551–0.747)	0.632(0.530–0.720)	0.696(0.528–0.857)	0.602(0.477–0.721)	0.450(0.311–0.596)	0.808(0.698–0.918)

Numbers in parentheses are the 95% confidence interval.

*DL* deep learning, *DLR* deep learning radiomic, *AUC* area under receiver operating characteristic curve, *ACC* accuracy, *SEN* sensitivity, *SPE* specificity, *PPV* positive predictive value, *NPV* negative predictive value.

### Radiomics, DL, DLR, and combined models performance and comparison

The radiomics model demonstrated an AUC of 0.731 (95% CI, 0.648–0.811) and an accuracy of 0.660 in the internal test cohort, respectively. In the external test cohort, the model achieved an AUC of 0.641 and an accuracy of 0.680. The DL model demonstrated limited generalizability, with AUCs of 0.561 (95% CI, 0.429–0.702) in the internal test cohort and 0.636 (95% CI, 0.522–0.745) in the external test cohort. The DLR model demonstrated moderate performance, with an AUC of 0.709 (95% CI, 0.595–0.813) and an accuracy of 0.635 in the internal test cohort. In the external test cohort, the model achieved an AUC of 0.707 (95% CI, 0.604–0.799) and an accuracy of 0.631.

The combined model yielded an AUC of 0.769 (95% CI, 0.662–0.864), with an accuracy of 0.728, a sensitivity of 0.885, a specificity of 0.658, a positive predictive value (PPV) of 0.535, and a negative predictive value (NPV) of 0.928 in the internal test set. In the external test cohort, the model exhibited an AUC of 0.771 (95% CI, 0.668–0.857), an accuracy of 0.722, a sensitivity of 0.752, a specificity of 0.708, a PPV of 0.547, and an NPV of 0.859.

The predictive performances of all models across cohorts are summarized in Table 2. The predictive performances of various models, along with radiologist assessments, were compared using the DeLong test (Supplementary Table 2). The combined model exhibited the highest predictive performance across all datasets and showed a statistically significant improvement over the other models in the external test cohort (DeLong test, all *p* < 0.05).

### Clinical utility

**Fig. 3:**
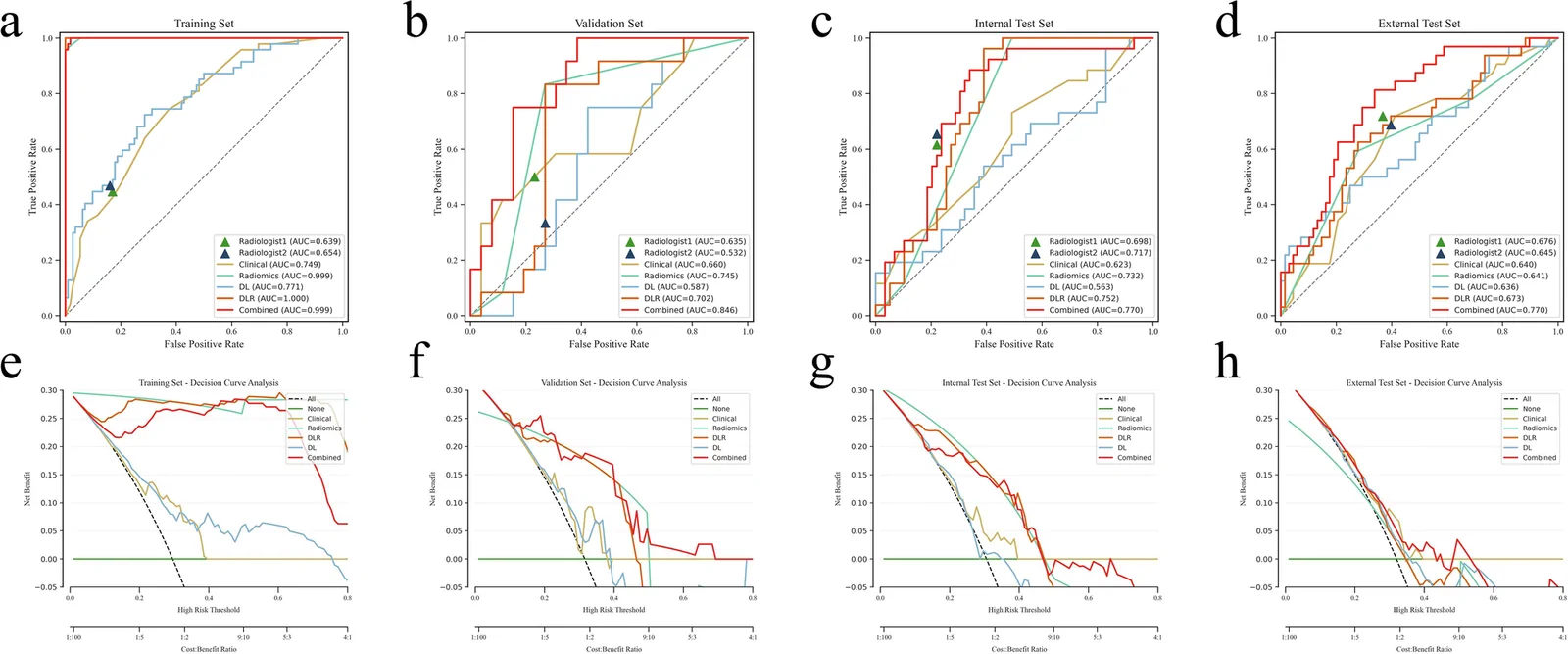
Model performance across cohorts. **a**–**d** Receiver operating characteristic (ROC) curves of clinical, radiomics, deep learning (DL), deep learning–radiomics (DLR), and multimodal combined models in the training, internal validation, internal test, and external validation cohorts. **e**–**h** Decision curve analyses (DCA) showing the net clinical benefit of each model across threshold probabilities (0–1). The multimodal combined model demonstrated the highest net benefit in the clinically relevant range (0.2–0.6).

When comparing the clinical, radiomics, DL and DLR models individually, the combined model demonstrated the highest net benefit across most threshold probabilities and consistently outperformed the “Treat All” and “Treat None” strategies in all four cohorts (Fig. 3). In the context of clinical decision-making, the combined model provided a greater net clinical benefit in a wide range of risk thresholds.

### Model calibration and probability prediction performance (brier score)

The calibration performance of the models was evaluated using calibration curves and Brier scores. The calibration curves of the combined models demonstrated good agreement between predicted probabilities and observed outcomes in both internal and external cohorts, with no evidence of systematic over- or underestimation across the risk spectrum (Supplementary Fig. 1).

Table 3 illustrated the probability prediction performance of the developed models, quantified by the Brier score. In the external test cohort, the combined model demonstrated robust performance with a Brier score of 0.217 (95% CI: 0.200–0.234), which was superior to the radiomics (0.274) and DL models (0.277), as well as radiologist assessment (ranging from 0.340 to 0.371). Although the training cohorts exhibit low Brier scores, the higher Brier scores observed in the validation and internal/external test cohorts indicated more realistic probability estimates under multicenter conditions.

**Table 3:** Probability prediction performance (brier score) of models across cohorts

Model	Train cohort (95%CI)	Validation cohort (95%CI)	Internal test cohort (95%CI)	External test cohort (95%CI)
Clinical model	0.191(0.179–0.204)	0.196(0.172–0.220)	0.205(0.187–0.223)	0.210(0.194–0.226)
Radiomics model	0.021(0.016–0.028)	0.211(0.171–0.252)	0.225(0.197–0.254)	0.274(0.248–0.303)
DL model	0.199(0.187–0.213)	0.257(0.224–0.292)	0.258(0.236–0.279)	0.277(0.257–0.296)
DLR model	0.045(0.040–0.050)	0.191(0.159–0.225)	0.199(0.179–0.219)	0.240(0.221–0.260)
Combined model	0.051(0.045–0.058)	0.183(0.152–0.215)	0.189(0.169–0.210)	0.217(0.200–0.234)
Radiologist 1	0.289(0.256–0.320)	0.289(0.223–0.355)	0.270(0.228–0.311)	0.340(0.300–0.382)
Radiologist 2	0.295(0.263–0.325)	0.294(0.228–0.360)	0.258(0.216–0.301)	0.371(0.330–0.414)

### Model interpretability

**Fig. 4:**
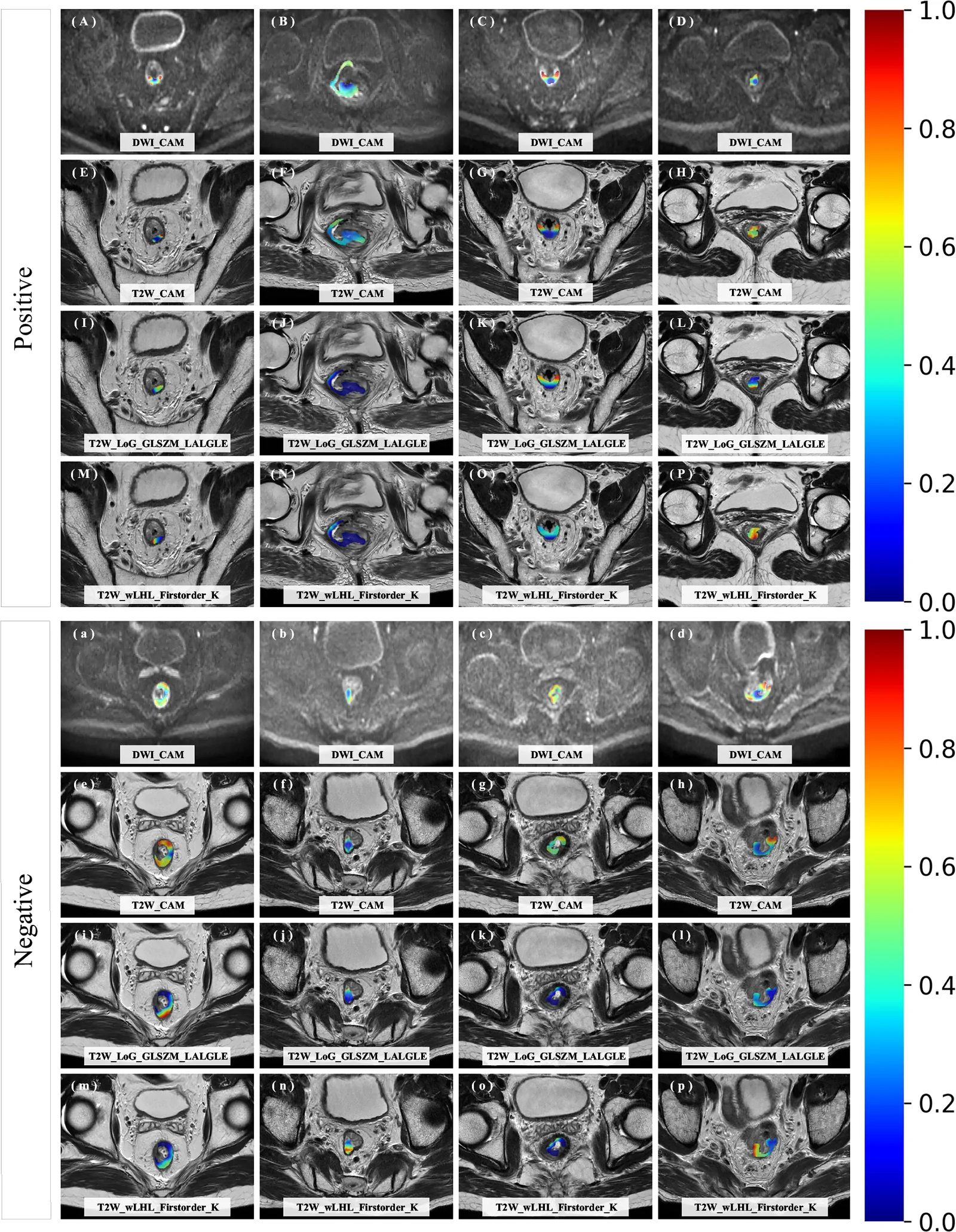
Example feature maps and Grad-CAM visualizations illustrating the top two radiomic features for four patients diagnosed with LNM and four patients with LARC without LNM. Grad-CAM images utilize a color gradient to depict normalized feature values, where blue and green indicate lower values, and red and yellow denote higher values. Regions at high risk for LNM exhibit elevated values in GLSZM_LargeAreaLowGrayLevelEmphasis and wavelet-LHL_firstorder_Kurtosis on T2WI. Panels (**A**–**H**; **a**–**h**) display Grad-CAM heatmaps that highlight deep features, with red and yellow areas indicating regions of high activation and blue and green areas indicating regions of low activation.

**Fig. 5:**
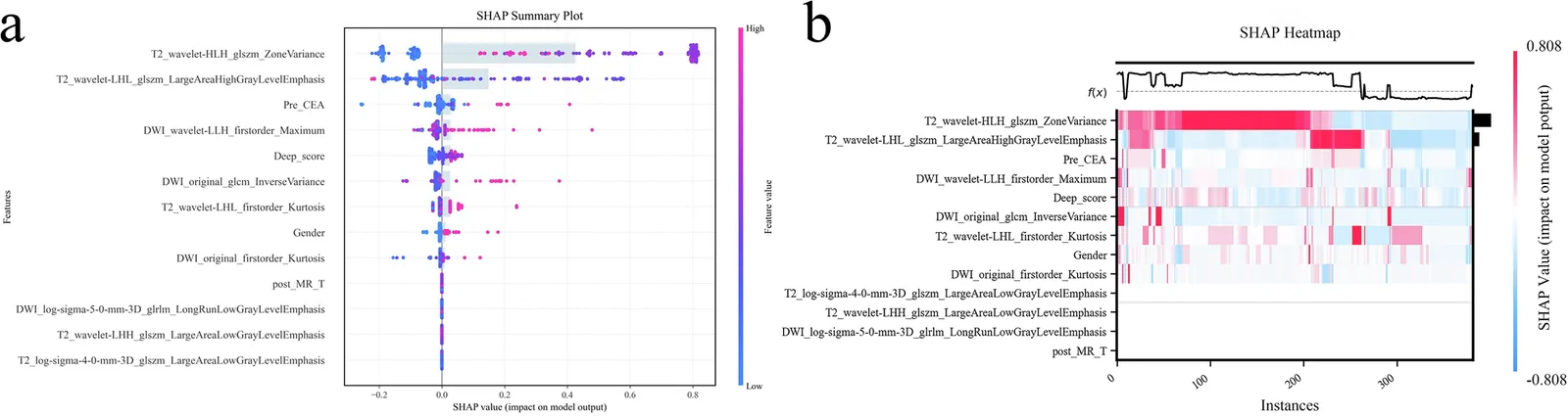
Interpretability analysis of the combined model using SHAP. **a** Global feature importance showed that T2-weighted texture features (reflecting tumor heterogeneity) and the DL score were the primary positive contributors to the predictions. The clinical variables followed the expected trends. **b** Force plot for an individual patient demonstrates how each feature shifts the model output from the base value towards the final prediction, illustrating the personalized decision-making process.

**Fig. 6:**
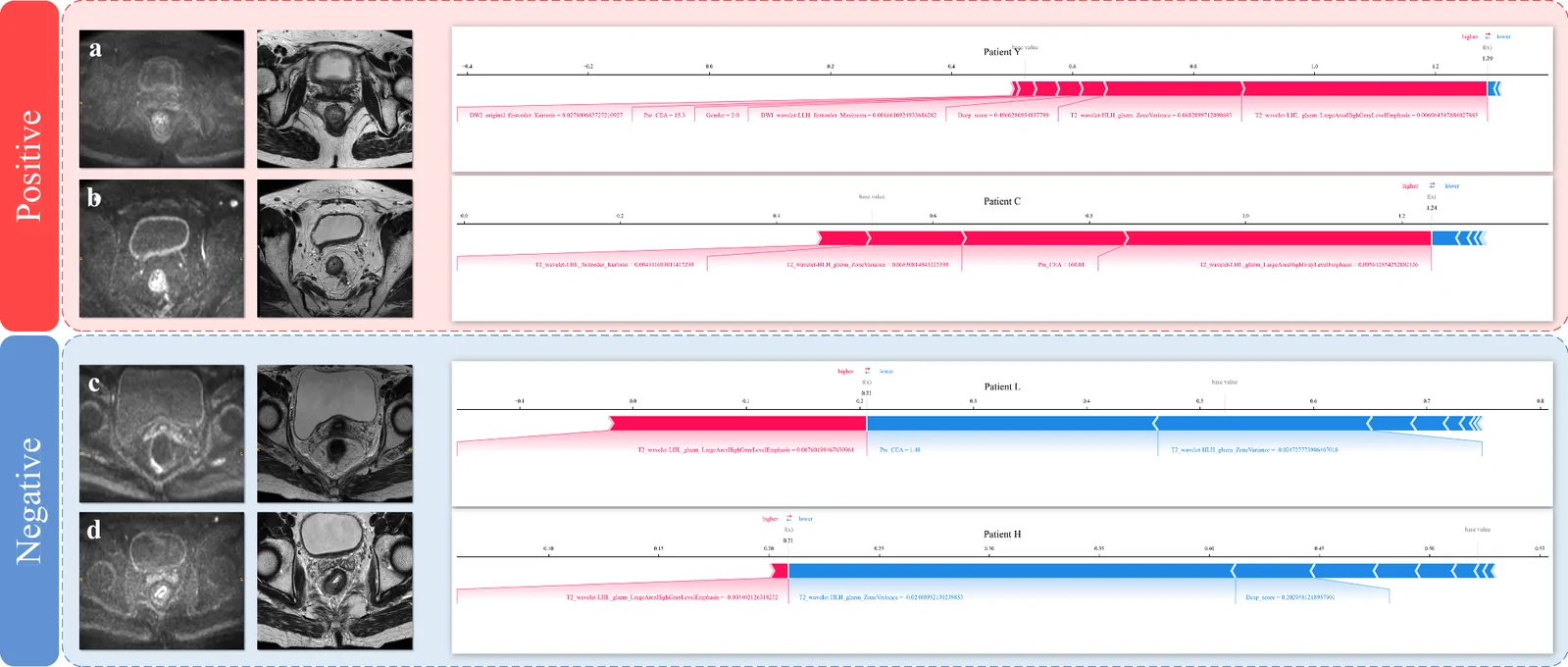
Case-level SHAP explanations. Patient 1 and Patient 2 demonstrated positive cases of lymph node invasion after nCRT correctly predicted by the model (**a**, **b**); Patient 3 and Patient 4 demonstrated the model’s correct prediction of negative cases of lymph node invasion after nCRT (**c**, **d**).

**Fig. 7:**
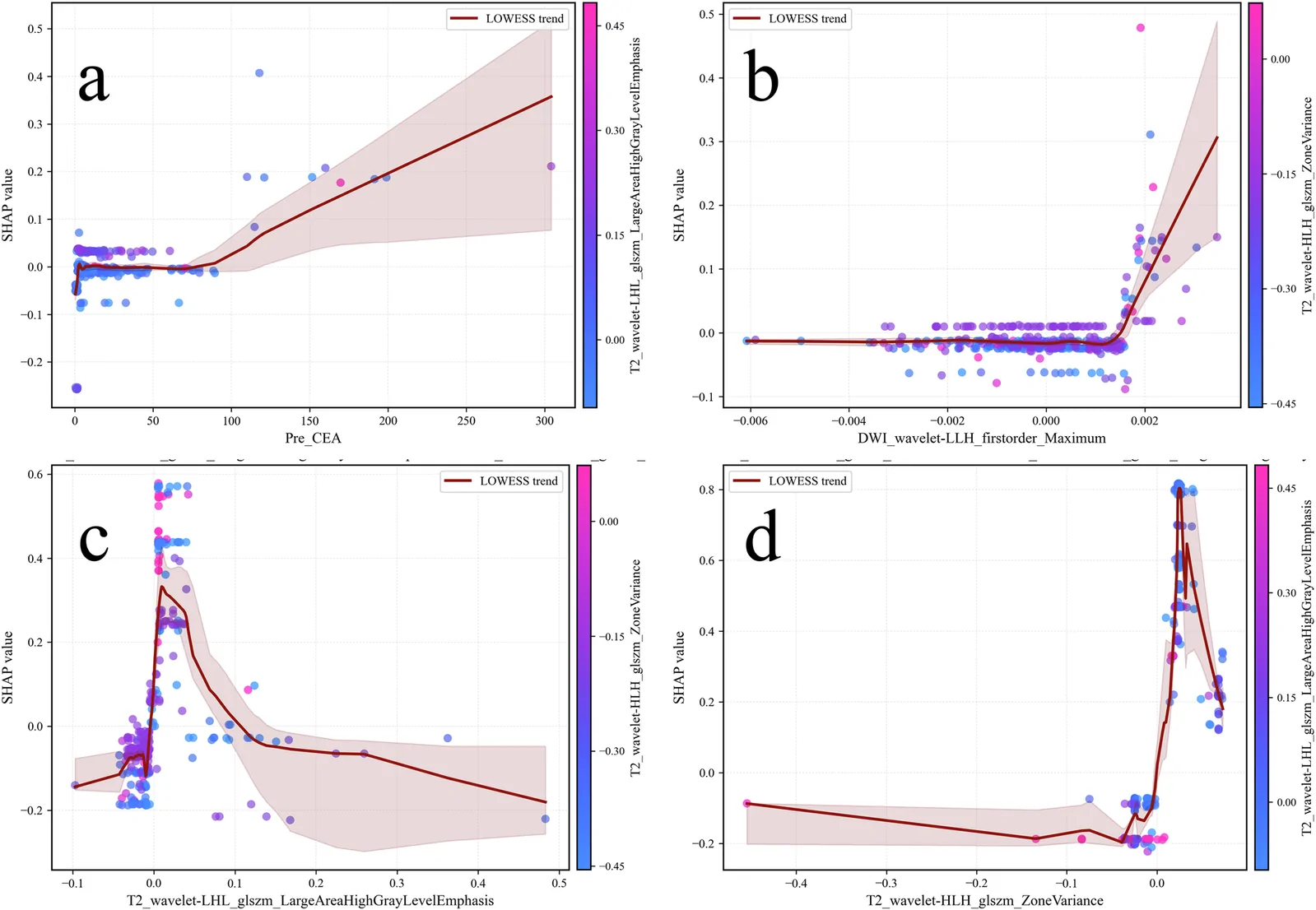
SHAP dependence plots for key multimodal features, revealing nonlinear effects and interactions. For each subplot, the x-axis shows the original feature value, the y-axis shows its impact on the prediction (SHAP value), and color represents the value of an interacting feature. The red curve is the smoothed trend (LOWESS). Analysis reveals: **a** a positive synergistic effect between a clinical and a radiomic feature; **b** a nonlinear response that is enhanced by interaction with another imaging feature; **c** peak predictive contribution at intermediate feature values; **d** highest sensitivity within a moderate range of heterogeneity.

Figures 4–7 present the interpretability analyses of the combined model. Grad-CAM visualizations (Fig. 4), which highlight areas in medical images that most influence model predictions, indicated that the deep-learning network primarily involved tumor margins and heterogeneous intratumoral regions. SHAP analyses (Figs. 5–7) further demonstrated that T2WI wavelet radiomics feature, pre-CEA level, DWI wavelet radiomics feature, and DL score were the most significant contributors to the combined model. Positive predictions at the individual-case level were primarily influenced by higher intratumoral heterogeneity, elevated DL scores, and increased pre-treatment CEA levels (Fig. 6). These interpretability results collectively provide biologically plausible explanations for model decisions, thereby enhancing the transparency and clinical credibility of the proposed framework.

## Discussion

In this multicenter study, we developed and externally validated an interpretable multiparametric MRI-based DLR model to predict LNM following nCRT in patients with LARC. The combined DLR model demonstrated stable performance in the external test cohort, achieving an AUC of 0.771 and producing well-calibrated probability estimates with a Brier Score of 0.217. DCA showed that the combined DLR model offered greater net clinical benefit compared to clinical, radiomics, DL, and DLR models and the assessments made by radiologists alone, across a clinically relevant risk threshold range of ~0.2–0.6. Our findings highlight the improved generalizability and clinical utility of this multimodal combined approach.

Accurate assessment of LNM after nCRT remains a significant diagnostic challenge in LARC management. A previous study indicated that post-nCRT LNM is characterized by scattered viable tumor cells within heterogeneous stromal environments instead of nodal enlargement^31^. Treatment-induced alterations, such as fibrosis, mucinous degeneration, necrosis, and inflammatory infiltration, often conceal residual tumor tissues, thereby limiting the accuracy of conventional MRI. The European Society of Gastrointestinal and Abdominal Radiology (ESGAR) recommends that a short-axis diameter greater than 5 mm be considered as positive lymph nodes for MR restaging^11^. Furthermore, Crimì et al. reported that measuring the long axis and volume of the largest mesorectal lymph node after nCRT may serve as a rapid and reliable alternative to ESGAR criteria for node restaging^32^. However, the cut-offs proposed in their study exhibited low sensitivity during validation.

Radiomics or DL models offer a noninvasive method for predicting response to nCRT. In this study, we found that radiomic texture features derived from T2WI and DWI substantially contributed to model predictions. These features capture intratumoral heterogeneity and microstructural complexity, reflecting residual tumor infiltration and treatment-related tissue remodeling^33,34^. Prior studies have similarly demonstrated the predictive value of multiparametric MRI-based radiomic signatures in assessing nodal status and treatment response in LARC^35,36^. Furthermore, DL-derived features complement handcrafted radiomics by encoding higher-order spatial information that is difficult to capture with predefined features alone^37,38^. The selected clinical factors comprised pre-treatment CEA levels and post-nCRT MRI-based T stage. Research has demonstrated that CEA enhances radio-resistance in the presence of M2 macrophages^39^ and elevated pre-treatment CEA levels are significantly associated with reduced response to nCRT and lower pCR rates^40,41^. Similarly, advanced ymrT stages indicate an increased probability of residual mural disease extending to mesorectal nodes^42^. By incorporating specific clinical variables, the model’s performance was improved and aligned better with existing clinical risk stratification frameworks.

Our interpretability analyses provide biological and imaging-based explanations for these findings. SHAP summary plot demonstrated that image-derived features and clinical indicators, particularly pre-nCRT CEA, collectively drove the risk estimates of LNM in the combined model. Wavelet- and texture-derived radiomics features from T2WI and DWI, together with DL score, were among the strongest contributors to model prediction. Wavelet features quantify gray-level heterogeneity and high-intensity distributions within the tumor, reflecting variations in structural organization and diffusion-related microstructural compactness. These features may enable the detection of subtle textural and structural differences that signify metastatic potential. In addition, the DL–derived score made a positive contribution, enhancing overall model stability and complementing radiomic features. Grad-CAM visualizations revealed that the network concentrated on tumor margins and heterogeneous intratumoral or peritumoral regions, which are pathologically relevant to residual tumor infiltration and perilymphatic spread. The observed spatial correspondence between model attention and pathologically relevant regions suggested that the model learns diagnostically meaningful representations rather than data-driven patterns. This dual XAI approach, which integrates global feature importance (SHAP) with local spatial explainability (Grad-CAM), represents a methodological best practice, offering a more interpretable, accurate and efficient prediction system^43^.

In addition to emphasizing discrimination, calibration is another essential issue that is frequently neglected in the clinical application of prediction models^44^. In this study, calibration curves and Brier scores confirmed that the combined model provided reasonably accurate probability estimates in both internal and external cohorts. DCA further showed that the model provides a net clinical benefit across a range of threshold probabilities, suggesting its potential as a decision-support tool for radiologist assessment.

This study had some limitations. First, its retrospective study design may introduce selection bias. Second, although we conducted an independent external test, the sample size remained limited. Third, despite cross-validation, external testing, and robust regularization, the near-perfect training AUC indicated the potential residual overfitting. Performance decreased to a more realistic level in the validation cohort, reflecting optimism bias during model development. Fourth, despite ComBat harmonization, variability in imaging protocols across centers may affect model performance. Fifth, the lack of molecular or genetic biomarkers limits interpretation of deep biological mechanisms. Therefore, large-scale, prospective, and more diverse validation studies integrating molecular and imaging data are needed to address these limitations.

In conclusion, this study developed and externally validated an interpretable multiparametric MRI-based combined model to predict LNM in patients with LARC following nCRT. By integrating clinical factors, radiomics, and DL representations, the proposed framework provides a quantitative supplement to traditional imaging and demonstrates preliminary cross‑institutional robustness, potentially facilitating individualized surgical strategies.

## Methods

### Study patients

The institutional review boards of all participating centers approved this multicenter retrospective study, and the need for written informed consent was waived. Furthermore, the study adhered to the STARD (Standards for Reporting of Diagnostic Accuracy Studies) guidelines^45^.

A total of 446 LARC patients, who underwent treatment between June 2017 and July 2023 at four tertiary medical centers, were retrospectively enrolled. All patients received standardized nCRT followed by total mesorectal excision. The inclusion criteria were as follows: (1) histologically confirmed rectal adenocarcinoma; (2) baseline MRI showing cT3–4 stage or node-positive disease; (3) completion of nCRT followed by TME within 6–8 weeks; (4) availability of complete clinicopathological data; and (5) high-quality, artifact-free MRI images. Patients who did not meet these criteria were excluded.

**Fig. 8:**
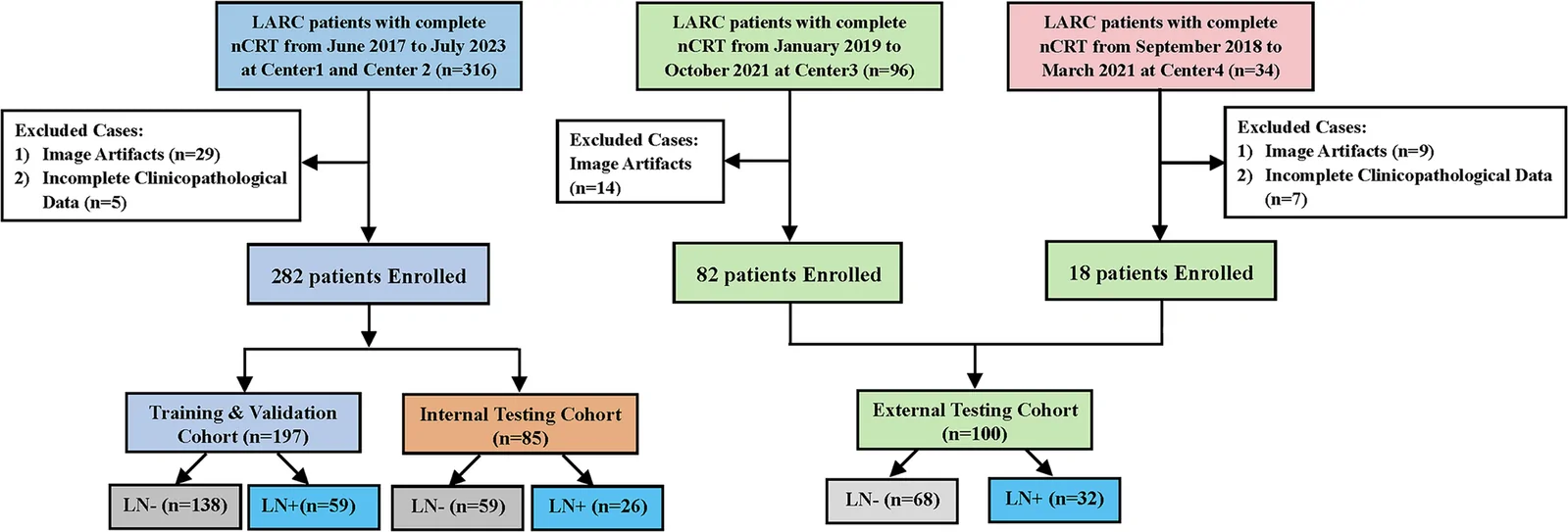
Flowchart of patient inclusion and cohort division.

Patients from Centers 1 and 2 were divided into a training set and an internal test set at a 7:3 ratio. During the training process, an internal validation set was split from the training set using 5-fold cross-validation, which is used specifically for model hyperparameter tuning. To ensure a strict and unbiased assessment of model generalizability, data from Centers 3 and 4 (*n* = 100) were completely held out and used exclusively as an independent external test cohort. These external data were not involved in any stage of model development, including feature selection, ComBat harmonization parameter estimation, hyperparameter tuning, or model training. The external test set was accessed only once after all model development procedures were finalized. A detailed patient inclusion flowchart is presented in Fig. 8.

### Clinical variables and reference standard

Clinical data, including patient’s age, gender, tumor location, post-nCRT clinical T stage, and pre-nCRT and post-nCRT CEA levels, were collected. The gold standard for determining LNM status is pathological analysis of surgically removed lymph node tissue.

### MRI acquisition and radiologist assessments

All patients underwent pelvic scanning with a 3.0 T MR scanner after nCRT and before surgery. The standard scanning protocols included high-resolution T2WI and DWI. The detailed imaging parameters are provided in Supplementary Table 3. Lymph node status was independently assessed by two experienced abdominal radiologists under blinded conditions, primarily based on conventional morphological criteria, such as short-axis diameter ≥ 6 mm, irregular edges, and heterogeneous signal intensity. In cases of discrepancies between the initial assessments, the findings were reviewed and resolved through a consensus discussion.

### Image preprocessing and segmentation

All images underwent standardized preprocessing, including N4 bias field correction to remove magnetic field inhomogeneities, voxel intensity normalization to 0–255, and spatial alignment with localized cropping centered on tumor boundaries. Two radiologists, each with 7 years of experience in rectal cancer imaging, manually outlined the entire tumor volumes on axial T2WI and DWI images after nCRT using ITK-SNAP software. To assess the stability of feature extraction, images from 45 randomly selected patients were analyzed. The intraclass correlation coefficient (ICC) was calculated for features extracted by the two radiologists. Only features with ICC values above 0.80 were retained to ensure robust inter-observer consistency.

### Radiomics feature extraction and selection

The open-source PyRadiomics toolkit was used to extract 1132 initial radiomics features separately from T2WI and DWI images, covering four categories: shape, first-order, texture, and transform features. To mitigate technical variability and batch effect associated with multicenter and different MR hardware, we applied the ComBat harmonization algorithm^46,47^. Importantly, the ComBat parameters were estimated solely from the training cohort (Centers 1 and 2) and then applied to transform the internal test and external test cohorts. This approach prevents batch‑effect correction from leaking information from the external validation set into model development.

To reduce overfitting and identify optimal features, the Least Absolute Shrinkage and Selection Operator (LASSO) regression was applied to the training cohort. The predictive performance of the selected features was validated on an independent dataset.

### DL feature extraction and multimodal fusion framework

To improve the model’s generalization, we developed a multimodal fusion framework. This framework integrates transfer learning, dual-stream encoders for T2WI and DWI, and auxiliary tasks for cross-modality alignment and classification. Imaging features were encoded via 3D ResNet50 initialized with Med3D pre-trained weights^48^. Clinical and radiomic features were processed using multilayer perceptron models. Subnetworks were jointly trained, and an attention-based pooling module was used to combine discriminative features for final classification. Two auxiliary tasks enhanced robustness: supervised contrastive learning for feature alignment and classification on concatenated imaging features. During inference, fused representations combined all modalities for improved accuracy. Grad-CAM highlighted predictive regions.

### Model establishment and evaluation

Following radiomics feature selection, a random forest (RF) classifier was constructed using the selected clinical and radiomic features. Hyperparameters for traditional machine learning and DL models were optimized separately to prevent information leakage. Hyperparameter tuning was conducted using five-fold cross-validation on the training cohort. For the RF model, grid search was performed to explore the number of estimators (50–500, step = 10), maximum tree depth (3–15), and minimum samples per leaf (1–5), with max_features set to auto. The number of radiomic features selected by LASSO was further tuned to 9–20. For the ResNet50-based DL model, random search (80 iterations) was applied to optimize the learning rate (1 × 10⁻⁵–5 × 10⁻³), batch size (16, 32, 48, 56), weight decay (1 × 10⁻⁶–1 × 10⁻²), and dropout rate (0.1–0.5). The loss function combined weighted binary cross-entropy with a supervised contrastive loss, with coefficients searched over 1–5. The network was initialized with pretrained weights and trained using stochastic gradient descent with momentum (0.9) and a piecewise linear learning rate decay schedule (see Supplementary Method). All hyperparameter tuning was restricted to the training cohort, and the optimal configuration was selected based on the highest AUC on the internal validation set. To ensure reproducibility, random seeds were fixed for Python, NumPy, PyTorch, and MONAI, and non-deterministic cuDNN operations were disabled. To avoid overfitting and ensure optimal performance, hyperparameter tuning was performed using a 5-fold cross-validation strategy on the training set.

Five prediction models were developed and compared in this study: (1) a clinical model utilizing only clinical variables; (2) a radiomics model based on filtered radiomics features; (3) a DL model based only on DL image features; (4) a DLR model integrating DL and radiomics features; and (5) a multimodal combination model. The DLR model is the direct output of the above-mentioned end-to-end multimodal fusion framework. The combined model was constructed using a bagging integration strategy, which computed a weighted average of the prediction probabilities from both the clinical model and the multimodal DLR model.

Predictive performance was evaluated using the area under the receiver operating characteristic curve (AUC); model AUCs were compared with the DeLong test. Calibration was evaluated via calibration curves and Brier scores. Clinical utility was measured through decision curve analysis (DCA) to quantify the net benefit of predictive models. To improve interpretability, the SHAP framework was applied to analyze feature contributions. Dependency graphs were combined with Grad-CAM heatmaps to visualize key features and the model’s decision rationale.

### Statistical analysis

All statistical analyses were performed using SPSS software (version 26.0; IBM, New York, USA). Continuous variables were compared using the independent-samples *t*-test or Mann–Whitney *U* test, while categorical variables were analyzed using the chi-square or Fisher’s exact test, as appropriate. A two-sided *P*-value < 0.05 was considered statistically significant.

## Supplementary information

Supplementary information

## Data Availability

The datasets generated and/or analyzed during the current study are available from the corresponding author on reasonable request.

## Code availability

All code, preprocessing pipelines, and trained model weights are available upon reasonable request for research reproducibility.
