# Supplementary material for: A multimodal interpretable deep learning-radiomics framework for predicting lymph node metastasis following neoadjuvant chemoradiotherapy in locally advanced rectal cancer: a multicenter validation study
Source: NPJ Precis Oncol. 2026 May 29;10:327. doi: 10.1038/s41698-026-01510-1 (PMC13503755; doi:10.1038/s41698-026-01510-1)
Supplement: Supplementary file 1 [file 41698_2026_1510_MOESM1_ESM.docx]

**Supplementary Materials**

**Supplementary Table 1 Comparison of Baseline Clinical Features between Center 3 and Center 4**

| **Characteristics** | **Center 3(n = 18)** | **Center 4(n = 82)** | **P Value*** |
| --- | --- | --- | --- |
| Age (y), mean ± SD | 56.67±9.50 | 55.31±11.13 | 0.634 |
| Gender (%) |  |  |  |
| Male | 15(83.3 %) | 56(68.29%) | 0.260 |
| Female | 3(16.7%) | 26(31.71%) |  |
| Chemotherapy Regimen (%) |  |  | 1.000 |
| Capeox | 15( 83.3%) | 62(75.6%) |  |
| Capecitabine | 1(5.6%) | 6(7.3%) |  |
| mFOLFOX6 | 2(11.1%) | 10(12.2%) |  |
| 5-fluorouracil and leucovorin | 0(0%) | 4(4.9%) |  |
| Post MR_T stage (%) |  |  | ＜0.001* |
| T1 | 0(0 %) | 0(0%) |  |
| T2 | 4(22.2%) | 4(4.9%) |  |
| T3 | 13(72.2%) | 24(29.3%) |  |
| T4 | 1 (5.6%) | 54(65.8%) |  |
| Tumor Location (%) |  |  | 0.874 |
| Low | 5(27.8%) | 25(13.4 %) |  |
| Middle | 10(55.6%) | 46( 30.4%) |  |
| Upper | 3(16.6%) | 11( 50.6%) |  |
| ypT Stage (%) |  |  | ＜0.001* |
| T1 | 0( 0%) | 1(1.2%) |  |
| T2 | 0 ( 0%) | 10(12.2%) |  |
| T3 | 17( 94.4%) | 11(13.4%) |  |
| T4 | 1(5.6%) | 60(73.2%) |  |
| yTRG(%) |  |  | 0.532 |
| TRG 0 | 4(22.2%) | 18 (21.9 %) |  |
| TRG 1 | 5(27.8%) | 20( 24.3%) |  |
| TRG 2 | 8(44.4%) | 43(52.4%) |  |
| TRG 3 | 1(5.6%) | 1(1.2%) |  |
| Histological Grade (%) |  |  | 0.047* |
| Well differentiation | 0(0%) | 11(13.4%) |  |
| Moderate differentiation | 18(100%) | 60(73.2%) |  |
| Poor differentiation | 0(0%) | 11(13.4%) |  |
| Pre-nCRT CEA Level (%) |  |  | 0.790 |
| ≤5(Normal) | 8(44.4%) | 31(37.8%) |  |
| >5(Abnormal) | 10 (55.6%) | 51(62.2%) |  |
| Post-nCRT CEA Level (%) |  |  | 0.268 |
| ≤5(Normal) | 15(83.3%) | 57(69.5%) |  |
| >5(Abnormal) | 3(16.7%) | 25(30.5%) |  |
| ypN Stage |  |  | 0.785 |
| ypN0 | 13 (72.2%) | 55 (67.1%) |  |
| ypN+ | 5 (27.8%) | 27(32.9%) |  |

Abbreviations: LN, lymph node; nCRT, neoadjuvant chemotherapy; CEA, carcinoembryonic antigen.

Note: Chi-squared or Fisher's exact tests, were used to compare the differences in categorical variables, whereas student t or Mann-Whitney U test was used to compare the differences in continuous variables, as appropriate.

**Supplementary Table 2 Performance comparison between different models in the training, validation, internal and external test sets.**

| **Training Set** | Clinical Model | Clinical Model | Radiomics Model | DL Model | DLR Model | Combined Model |
| --- | --- | --- | --- | --- | --- | --- |
|  | Clinical Model | 1.000 | <0.001 | 0.383 | <0.001 | <0.001 |
|  | Radiomics Model | <0.001 | 1.000 | <0.001 | 0.780 | 0.984 |
|  | DL Model | 0.383 | <0.001 | 1.000 | <0.001 | <0.001 |
|  | DLR Model | <0.001 | 0.780 | <0.001 | 1.000 | 0.367 |
|  | Combined Model | <0.001 | 0.984 | <0.001 | 0.367 | 1.000 |
| **Internal Validation Set** | Clinical Model | Clinical Model | Radiomics Model | DL Model | DLR Model | Combined Model |
|  | Clinical Model | 1.000 | 0.122 | <0.001 | 0.148 | <0.001 |
|  | Radiomics Model | 0.122 | 1.000 | <0.001 | 0.179 | 0.089 |
|  | DL Model | <0.001 | <0.001 | 1.000 | <0.001 | <0.001 |
|  | DLR Model | 0.148 | 0.179 | <0.001 | 1.000 | <0.001 |
|  | Combined Model | <0.001 | 0.089 | <0.001 | <0.001 | 1.000 |
| **Internal Test Set** | Clinical Model | Clinical Model | Radiomics Model | DL Model | DLR Model | Combined Model |
|  | Clinical Model | 1.000 | 0.002 | 0.049 | 0.001 | <0.001 |
|  | Radiomics Model | 0.002 | 1.000 | <0.001 | 0.356 | 0.119 |
|  | DL Model | 0.049 | <0.00 | 1.000 | <0.001 | <0.001 |
|  | DLR Model | 0.001 | 0.356 | <0.001 | 1.000 | 0.001 |
|  | Combined Model | <0.001 | 0.119 | <0.001 | 0.001 | 1.000 |
| **External Test Set** | Clinical Model | Clinical Model | Radiomics Model | DL Model | DLR Model | Combined Model |
|  | Clinical Model | 1.000 | 0.987 | 0.925 | 0.079 | 0.002 |
|  | Radiomics Model | 0987 | 1.000 | 0.944 | 0.137 | <0.001 |
|  | DL Model | 0.925 | 0.944 | 1.000 | <0.001 | <0.001 |
|  | DLR Model | 0.079 | 0.137 | <0.001 | 1.000 | <0.001 |
|  | Combined Model | 0.002 | <0.001 | <0.001 | <0.001 | 1.000 |

Note. DL, deep learning; DLR, deep learning radiomic.

**Supplementary Fig 1. Calibration curves of the training set, validation set, internal test set, and external test set. Lines of different colors represent the discriminative ability of different models. B represents the Brier score.**

**Supplementary Table 3. MRI sequences parameters of T2WI and DWI of different MR devices**

| **Parameters** | **Center 1** | | **Center 2** | | | | | | **Center 3** | | **Center 4** | |
| --- | --- | --- | --- | --- | --- | --- | --- | --- | --- | --- | --- | --- |
|  | **Philips Achieve 3.0 T** | | **Siemens Verio 3.0T** | | **Philips Ingenia 3.0T** | | **Siemens Prisma 3.0T** | | **Discovery 750w 3.0T** | | **Siemens Verio 3.0T** | |
| MR Sequence | T2WI | DWI | T2WI | DWI | T2WI | DWI | T2WI | DWI | T2W | DWI | T2W | DWI |
| Repetition time (ms) | 3906 | 2000 | 6350 | 5900 | 3664 | 3514 | 8040 | 4900 | 2690 | 4600 | 4200 | 4455 |
| Echo Time (ms) | 100 | 60 | 93 | 83 | 100 | 81 | 89 | 53 | 76 | 84 | 102 | 105 |
| Flip Angle (°) | 90 | 90 | 140 | 90 | 90 | 90 | 160 | 90 | 90 | 90 | 115 | 90 |
| Imaging Frequency (Hz) | 127.8 | 127.8 | 123.2 | 123.2 | 127.8 | 127.8 | 123.2 | 123.2 | 123.2 | 123.2 | 127.7 | 127.7 |
| Number of Average | 1 | 2 | 2 | 2 | 2 | 2 | 1 | 1 | 2 | 2 | 1.5 | 1 |
| Percent Sampling (%) | 100 | 100 | 80 | 80 | 84 | 99 | 80 | 100 | 150 | 75 | 100 | 100 |
| Pixel Bandwidth | 218 | 2567 | 260 | 1628 | 325 | 2303 | 200 | 1985 | 199 | 1163 | 244 | 1953 |
| Matrix | 316×314 | 120×118 | 320×256 | 192×115 | 288×228 | 108×106 | 320×240 | 140×104 | 256×215 | 172×111 | 288×256 | 128×128 |
| Slice thickness (mm) | 3 | 4 | 3 | 5 | 3 | 4 | 3 | 4 | 3 | 5 | 4 | 5 |

**Supplementary Method. The training process of the model**

**Deep Learning Architecture and Feature Extraction**

To quantitatively assess tumor phenotypes and enhance the multimodal predictive model, we have developed a comprehensive deep learning (DL) framework. This framework encompasses feature extraction methodologies and training parameters, thereby ensuring reproducibility and technical transparency. The DL architecture employs a multimodal fusion design that integrates dual-stream image encoders, clinical and radiomics encoders, cross-modal feature alignment, and an attention-based fusion module. The backbone of this architecture is a 3D ResNet50, as depicted in Fig. 2. Specifically, the dual-stream image encoder processes T2-weighted (T2W) and diffusion-weighted (DW) MRI, while the multilayer perceptron (MLP) encoders handle clinical and radiomics features. The attention module subsequently fuses all features to facilitate the final classification.

**Dual-Stream Image Encoder**

The dual-stream encoder is designed to extract high-dimensional features from T2-weighted (T2W) and diffusion-weighted (DW) MRI sequences. Tumor regions of interest (ROIs) from a cohort of 382 patients were subjected to preprocessing and resampling, standardizing them to a uniform size of 48×48×48 voxels to maintain consistent input dimensions. Each stream utilizes a 3D ResNet50 architecture, which begins with an initial 7×7×7 3D convolution layer, followed by four residual stages. Each stage is composed of multiple BasicBlocks, each containing two 3×3×3 convolutional layers and shortcut connections to facilitate residual learning. The encoder maps the extracted features into a 256-dimensional space and incorporates an independent classification head to enable single-modality training.

**Clinical and Radiomics Feature Encoders**

The clinical and radiomics features are encoded through distinct multilayer perceptrons (MLPs). Each encoder comprises an input layer, multiple residual modules, and a classification layer, culminating in the production of 256-dimensional feature representations. The clinical encoder is specifically optimized for low-dimensional inputs by employing layer normalization and a gradual expansion of feature dimensions. Conversely, the radiomics encoder, tailored for high-dimensional data, integrates both batch and layer normalization techniques with a progressive reduction in dimensionality.

**Multimodal Fusion and Attention Module**

All encoders, with the exception of the dual-stream image encoders, were initialized using random weights. The dual-stream image encoders were initialized with Med3D pretrained weights derived from 23 datasets. Initially, each encoder underwent pretraining specific to its respective modality. Subsequently, they were jointly optimized within a multimodal framework. An attention pooling fusion module concatenates the four 256-dimensional feature vectors into a 4×256 representation and processes them using eight attention heads. This mechanism automatically assesses the significance of each modality, thereby generating a fused 256-dimensional feature vector for classification purposes.

**Cross-Modal Feature Alignment**

Two tasks were executed to improve modality integration:

1. Cross-modal feature alignment, which involved the application of supervised contrastive loss to decrease similarity between negative pairs while increasing similarity between positive pairs. This approach facilitates modality-invariant feature learning and enhances robustness against diverse imaging or clinical protocols.
2. Image modality classification, where the concatenated outputs of the dual-stream encoders were directly utilized for classification, thereby highlighting the significance of imaging features.

**Data Augmentation**

Data augmentation was conducted utilizing the MONAI library. The spatial augmentations implemented included random flipping with a probability of 0.5, affine transformations comprising translations of ±1 voxel, rotations of ±10°, and scaling of ±10%, all applied with a probability of 0.8, as well as random 3D elastic deformations characterized by a sigma range of 2–3 and a magnitude range of 5–10, with a probability of 0.5. Intensity augmentations were tailored to specific modalities: T2-weighted (T2W) images underwent random contrast adjustments with a gamma range of 0.8–1.2 and a probability of 0.5, while diffusion-weighted (DW) images were subjected to Gaussian noise with a mean of 0 and a standard deviation of 0.03, also with a probability of 0.5. These augmentation strategies are designed to enhance the model's robustness against variations in imaging conditions.

**Training Strategy**

The model underwent training utilizing stochastic gradient descent (SGD) with a batch size of 48 over the course of 80 epochs. The overall loss function was composed of two weighted binary cross-entropy (BCE) components for classification purposes, alongside a symmetric supervised InfoNCE loss for feature alignment, with a temperature parameter set at 0.7. The learning rate was initially set at 5×10⁻³ and was adjusted linearly upwards during the initial 12 epochs. It then decreased linearly until epoch 50, after which it oscillated every ten epochs for a total of three cycles, in order to mitigate the risk of convergence to local minima.

**Implementation Environment**

The training process was conducted utilizing an NVIDIA RTX 2080Ti GPU equipped with 11 GB of memory. The software stack comprised PyTorch version 1.10, MONAI version 1.3, and Python version 3.8.
